# Insulin Resistance-Related Traits and Diabetic Maculopathy: Causal Insights from Mendelian Randomization

**DOI:** 10.3390/biomedicines14061178

**Published:** 2026-05-22

**Authors:** Young Lee, Je Hyun Seo, Sung Pyo Park

**Affiliations:** 1 Veterans Medical Research Institute, Veterans Health Service Medical Center, Seoul 05368, Republic of Korea; lyou7688@gmail.com; 2 Department of Ophthalmology, Veterans Health Service Medical Center, Seoul 05368, Republic of Korea; 3 Department of Ophthalmology, Kangdong Sacred Heart Hospital, Hallym University College of Medicine, Seoul 05355, Republic of Korea; eyepyo@gmail.com

**Keywords:** body mass index, diabetic maculopathy, Mendelian randomization, insulin-like growth factor 1, insulin resistance

## Abstract

**Background/Objectives:** To investigate the causal relationships linking body mass index (BMI) and circulating insulin-like growth factor 1 (IGF-1) levels with diabetic maculopathy risk using two-sample Mendelian randomization (MR). **Methods**: A two-sample MR framework was applied, utilizing genetic instruments for BMI and IGF-1 derived from the UK Biobank. Summary-level diabetic maculopathy data were obtained from the FinnGen consortium. Genome-wide significant single-nucleotide polymorphisms (SNPs, *p* < 5.0 × 10^−8^) independently associated with each exposure were employed as instrumental variables. Primary causal estimates were obtained using the inverse-variance weighted (IVW) method. Sensitivity analyses, including MR-Egger regression, weighted median methods, and the MR-Pleiotropy RESidual Sum and Outlier (MR-PRESSO), were conducted to evaluate robustness and potential pleiotropy. **Results:** Genetically predicted BMI was positively associated with diabetic maculopathy risk in both the IVW analysis (odds ratio [OR] = 1.16 (95% confidence interval [CI]: 1.04–1.30), *p* = 0.008) and MR-PRESSO (OR = 1.16 (95% CI: 1.04–1.28), *p* = 0.006). MR-PRESSO exhibited a significant relationship between higher IGF-1 levels and increased diabetic maculopathy risk (OR = 1.09 (95% CI: 1.01–1.18), *p* = 0.025), whereas the IVW method indicated only a suggestive association (OR = 1.08 (95% CI: 0.99–1.18), *p* = 0.087). **Conclusions:** The genetic evidence supports a causal role of insulin resistance-related traits in diabetic maculopathy development, with higher BMI and IGF-1 levels increasing diabetic maculopathy risk. These results underscore the potential contributory role of IGF-1 in disease pathogenesis and suggest that insulin resistance-related traits may represent preventive therapeutic targets.

## 1. Introduction

Diabetes mellitus (DM) affected approximately 10.5% of individuals aged 20–79 years worldwide in 2021, and the rate is projected to increase to 12.2% by 2045. Among the complications associated with DM, diabetic retinopathy represents the primary ocular manifestation [1]. Diabetic maculopathy, commonly referred to as diabetic macular edema (DME), is a principal contributor to vision impairment globally [2]. A meta-analysis based on data from 22,896 individuals with DM reported prevalences of 10.2% and 6.81% for diabetic retinopathy and diabetic maculopathy, respectively [3]. Diabetic maculopathy is characterized by retinal thickening and hard exudates in the posterior pole, driven by the accumulation of intraretinal fluid principally within the inner and outer plexiform layers [4]. The outcomes of diabetic maculopathy vary depending on whether treatment is initiated in a timely manner, with early interventions often reversing vision loss; conversely, chronic untreated disease may result in irreversible damage and permanent blindness. Thus, from a clinical perspective, elucidating the precipitating factors underlying diabetic maculopathy is essential for advancing the pathophysiologic understanding of the disease and enabling the development of novel therapeutic and preventive strategies to combat recurrence.

Diabetic maculopathy is a multifactorial condition involving metabolic dysregulation, vascular abnormality-induced angiogenesis, inflammation, and retinal neurodegeneration [5]. Among the systemic risk factors that have been identified to date, consistent evidence from epidemiological studies has established hyperglycemia as a key contributor to retinal edema [6,7,8], whereas adequate control of systemic hypertension has been shown to reduce the risk of diabetic retinopathy progression [6]. While several studies have examined the association between body mass index (BMI) and diabetic maculopathy, the reported findings remain inconsistent, and a definitive causal relationship has yet to be established. One observational study reported that more than 90% of patients with DME were overweight or obese [9]. In contrast, a recent study demonstrated that lower BMI and higher low-density lipoprotein cholesterol levels were associated with the occurrence of DME [10], whereas another study failed to identify a significant association between the development of DME and systemic metabolic parameters, including BMI, triglyceride, and high-density lipoprotein levels [11]. It is important to note that elevated insulin-like growth factor-1 (IGF-1) concentrations have been documented in the vitreous of patients with diabetic retinopathy [12], and IGF-1 has been shown to contribute to disease development by modulating vascular endothelial growth factor (VEGF) signaling via a phosphoinositide 3-kinase (PI3K)/protein kinase B (Akt)-dependent pathway involving hypoxia-inducible factor 1 alpha (HIF-1α) and nuclear factor kappa B (NF-κB)/activating protein 1 (AP-1) [13], thereby promoting vascular permeability and inducing inflammatory pathways [14,15]. Experimental studies have demonstrated that elevated intraocular IGF-1 levels can induce retinal vascular alterations resembling the changes observed in diabetic retinopathy, even in the absence of hyperglycemia [16]; however, clinical evidence remains inconsistent, suggesting that IGF-1 may act as a context-dependent modulator rather than a primary causal driver. Furthermore, a recent large-scale study documented an association between BMI and blood IGF-1 levels, highlighting the need to investigate the relationship between IGF-1 and BMI in this context [17].

Observational studies are inherently affected by confounding and selection bias, limiting the ability to accurately interpret the potential causal link between BMI or circulating IGF-1 levels and the occurrence of diabetic maculopathy. These limitations can be overcome using alternative approaches such as Mendelian randomization (MR), a method that leverages genetic variants associated with an exposure of interest as instrumental variables (IVs) to strengthen causal inference [18,19]. Because genetic variants are randomly allocated at conception, MR analyses are less prone to confounding and reverse causation, and several recently published studies on risk factor analysis in ophthalmological research have effectively employed MR techniques [20,21,22,23,24]. Therefore, the primary endpoint of this study was to evaluate the causal effect of BMI on the risk of diabetic maculopathy using a two-sample MR framework based on genome-wide association study (GWAS) data. As a secondary endpoint, we investigated the potential causal role of circulating IGF-1 levels, another key insulin resistance-related trait, in the occurrence of diabetic maculopathy within the same analytical framework.

## 2. Materials and Methods

### 2.1. Study Design

Our research adhered to the standards of the Declaration of Helsinki, and the protocol received approval from the Institutional Review Board of the Veterans Health Service Medical Center (IRB No. 2026-01-034), with the requirement for informed consent waived in light of the retrospective design.

### 2.2. Data Sources

**Figure 1:**
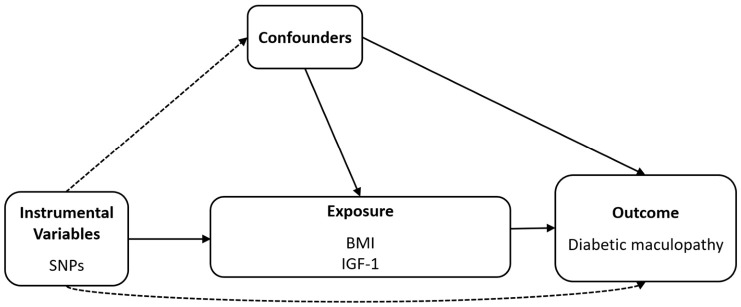
Conceptual framework of the variables applied in the two-sample Mendelian randomization design. Solid lines indicate required associations; dashed lines indicate prohibited associations under the Mendelian randomization assumptions. BMI, body mass index; IGF-1, insulin-like growth factor 1; SNP, single-nucleotide polymorphism.

A graphical overview of the study’s analytical framework is presented (Figure 1). To investigate the causal effects of BMI and IGF-1 levels on the risk of diabetic retinopathy, the following datasets were used: (1) exposure data collected from the summary statistics of the UK Biobank GWAS (*n* = 413,186 for BMI; *n* = 398,797 for IGF-1; https://pan.ukbb.broadinstitute.org/downloads, accessed on 20 February 2026, Table 1); and (2) outcome data collected from the summary statistics of the FinnGen GWAS [*n* = 86,890 (4603 cases and 82,287 controls); https://finngen.gitbook.io/documentation/data-download, accessed on 23 February 2026, Table 1].

### 2.3. Selection of the Genetic IVs

Instrumental variables were defined as SNPs fulfilling the genome-wide significance threshold (*p* < 5.0 × 10^−8^) for BMI and IGF-1 levels. To guarantee independence between the instrumental variables, SNPs were pruned according to linkage disequilibrium criteria (LD; r^2^ < 0.001, clumping distance = 10,000 kb). The 1000 Genomes Phase III Dataset (European population) was employed as the reference panel to estimate the LD for the clumping procedure. Each IV was appraised for strength through calculation of the F-statistic; more specifically, an F-statistic greater than 10 was considered indicative of sufficient instrument strength, suggesting a low risk of weak instrument bias [25].

### 2.4. MR Analyses

The MR analyses were undertaken under the subsequent core assumptions governing the validity of IVs: (1) they should provide evidence of a substantial connection with the exposure; (2) they should exhibit no relationship with confounding factors in the exposure–outcome pathway; and (3) they should influence the outcome only through the exposure, indicating the absence of a directional horizontal pleiotropy effect. Causal estimates were obtained using the inverse-variance weighted (IVW) method with multiplicative random effects as the primary analysis [26,27,28], in combination with complementary MR approaches, including the weighted median estimator method [29], MR-Egger regression with and without Simulation Extrapolation (SIMEX) correction [30,31], and the MR-Pleiotropy RESidual Sum and Outlier (MR-PRESSO) test [32]. IVW is widely recognized as the most robust method when all genetic variants comply with the three core MR criteria [33]; however, the results may be distorted when one or more variants are not valid instruments [29]. The weighted median method generates robust causal estimates even when up to 50% of the instrumental variables are invalid [29]. By allowing a non-zero intercept, the MR-Egger technique accounts for average horizontal pleiotropic effects and facilitates causal effect estimation in the presence of pleiotropy [30]. In cases where the no-measurement-error assumption is not met (*I*^2^ < 90%), MR-Egger estimates can be corrected using SIMEX [31]. MR-PRESSO identifies potential outliers and accounts for horizontal pleiotropy in the IVW analysis by excluding them [32]. Therefore, interpretation of the results was guided by the most fitting MR analytical framework [34]. Heterogeneity among IVs was assessed using Cochran’s Q statistic for the IVW analyses and Rücker’s Q′ statistic for the MR-Egger analyses [26,35]. Horizontal pleiotropy was evaluated using the MR-Egger intercept test and the MR-PRESSO global test. For Cochran’s Q statistic or Rücker’s Q′ statistic, *p* < 0.05 was considered indicative of the presence of heterogeneity among variant-specific causal estimates, whereas for the MR-Egger intercept test or MR-PRESSO global test, *p* < 0.05 was considered suggestive of horizontal pleiotropy. All causal effect estimates are reported as odds ratios (ORs) accompanied by 95% confidence intervals (CIs), and a two-tailed *p*-value of less than 0.05 was considered statistically significant. All analyses were performed using the TwoSampleMR and simex packages in R version 3.6.3 (R Core Team, Vienna, Austria).

## 3. Results

### 3.1. Genetic IVs in MR

Independent genetic variants associated with BMI (*n* = 467) and IGF-1 levels (*n* = 443) meeting the genome-wide significance criterion (*p* < 5.0 × 10^−8^) were identified and designated as IVs (Table 2). The mean F-statistics for these IVs were 61.31 for BMI and 107.01 for IGF-1, and each F-statistic exceeded the accepted threshold of 10, confirming that weak instrument bias was unlikely to affect the results (Table 2 and Table S1). Comprehensive details of the selected IVs are presented in Table S1. Heterogeneity was evaluated by applying Cochran’s Q statistic in the IVW framework and Rücker’s Q′ statistic in the MR-Egger approach. The results of Cochran’s Q test suggested the presence of heterogeneity for BMI and IGF-1 among the instruments (*p* < 0.001 and *p* = 0.005, respectively; Table 2); Rücker’s Q′ test also indicated significant heterogeneity for BMI and IGF-1 (*p* < 0.001 and *p* = 0.006, respectively; Table 2). Regarding horizontal pleiotropy, the MR-PRESSO global test suggested the presence of horizontal pleiotropic effects (*p* < 0.001 for BMI; *p* = 0.006 for IGF-1). In contrast, the MR-Egger intercept revealed no evidence of directional pleiotropy, either before (*p* = 0.330 for BMI; *p* = 0.299 for IGF-1) or after SIMEX correction (*p* = 0.288 for BMI; *p* = 0.309 for IGF-1). Based on the heterogeneity and potential pleiotropic influences, MR-PRESSO was used as the core MR analysis technique, and the IVW method with multiplicative random effects was adopted as a complementary approach [34].

### 3.2. MR for Assessing the Causal Effects of BMI and IGF-1 Levels on Diabetic Maculopathy

**Figure 2:**
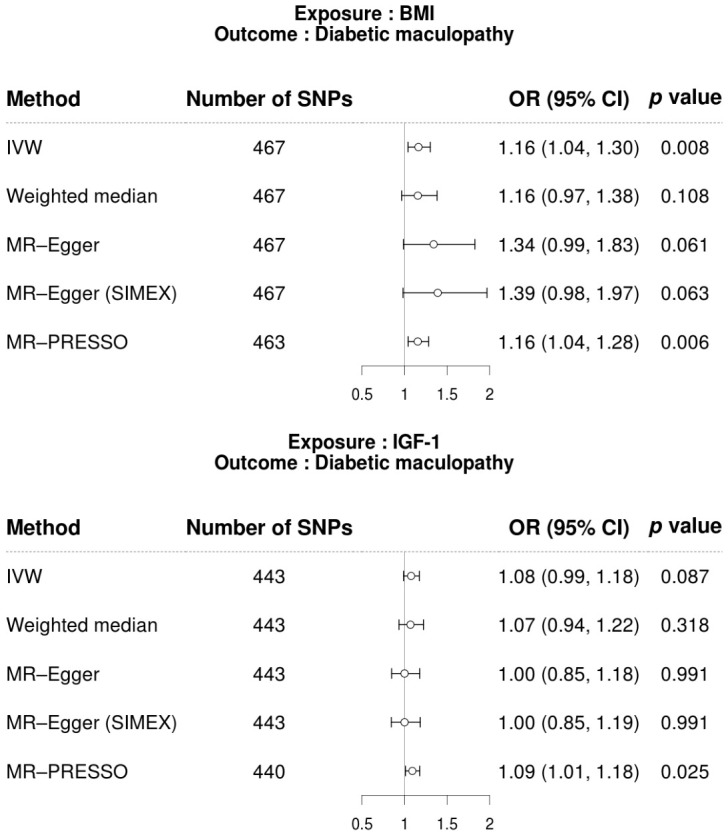
Forest plots of causal associations of BMI and IGF-1 with diabetic maculopathy. BMI, body mass index; CI, confidence interval; IGF-1, insulin-like growth factor 1; IVW, inverse-variance weighted; MR, Mendelian randomization; OR, odds ratio; PRESSO, Pleiotropy RESidual Sum and Outlier; SIMEX, simulation extrapolation; SNP, single-nucleotide polymorphism.

The MR analyses demonstrated that genetically predicted BMI was significantly associated with an increased risk of diabetic maculopathy. More specifically, the IVW method yielded an OR of 1.16 (95% CI: 1.04–1.30, *p* = 0.008), which was consistent with the MR-PRESSO results (OR = 1.16 (95% CI: 1.04–1.28), *p* = 0.006). The MR-Egger analysis revealed a similar direction of effect without reaching statistical significance (OR = 1.34 (95% CI: 0.99–1.83), *p* = 0.061). The results remained consistent after SIMEX correction (OR = 1.39 (95% CI: 0.98–1.97), *p* = 0.063). As shown in Figure 2, the causal estimates were generally consistent in direction across the different MR methods used in the study. The lack of statistical significance in the MR-Egger analysis may reflect its relatively lower statistical power compared with that of other MR approaches [30].

**Figure 3:**
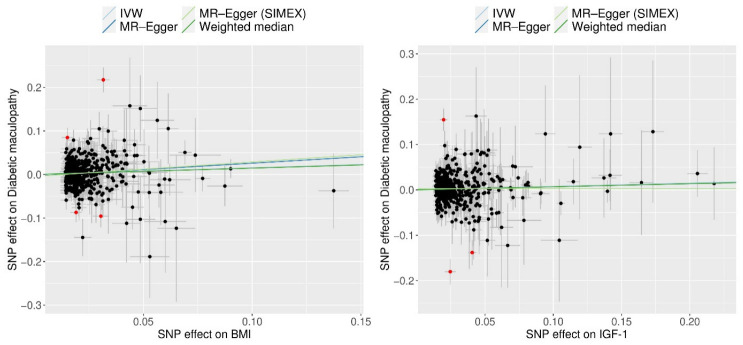
Scatter plots of the MR tests used to assess the effects of BMI and IGF-1 levels on the occurrence of diabetic maculopathy. Light blue, dark blue, light green, and dark green regression lines represent the IVW, MR-Egger, MR-Egger (SIMEX), and weighted median estimates, respectively. The slope of each line represents the causal effect calculated using each method. Each dot corresponds to an SNP, with the x-axis representing the association between the SNP and the exposure and the y-axis representing the association between the SNP and the outcome. Red dots indicate outliers in the MR-PRESSO analysis. BMI, body mass index; IGF-1, insulin-like growth factor 1; IVW, inverse-variance weighted; MR, Mendelian randomization; PRESSO, Pleiotropy RESidual Sum and Outlier; SIMEX, simulation extrapolation; SNP, single-nucleotide polymorphism.

Increased circulating IGF-1 levels were significantly linked to a higher risk of diabetic maculopathy in the MR-PRESSO analysis (OR = 1.09 (95% CI: 1.01–1.18), *p* = 0.025), whereas the MR analysis based on the IVW method demonstrated only a suggestive correlation (OR = 1.08 (95% CI: 0.99–1.18), *p* = 0.087). The SNP-specific associations of BMI and IGF-1 with diabetic maculopathy are illustrated in a scatter plot (Figure 3).

## 4. Discussion

Optimal systemic control—particularly stringent regulation of hyperglycemia, hypertension, and dyslipidemia—is fundamentally important in delaying the onset and progression of diabetic retinopathy and diabetic maculopathy; however, the role of modifiable systemic biomarkers in the monitoring and management of diabetic maculopathy remains insufficiently defined. Moreover, the mechanistic contribution of metabolic dysfunction to diabetic maculopathy development is poorly understood, underscoring a critical knowledge gap. The results of this two-sample MR study demonstrated that the genetically predicted BMI was significantly associated with an elevated diabetic maculopathy risk. In addition, circulating IGF-1 levels exhibited a consistent direction of effect across the analytical methods employed, reaching statistical significance in the MR-PRESSO analysis and a suggestive association based on the IVW method. These findings support a potential causal role of metabolic factors, particularly adiposity and growth factor signaling, in the pathogenesis of diabetic maculopathy.

The observed association between BMI and diabetic maculopathy is biologically plausible, as obesity has been shown to be closely linked to insulin resistance, chronic low-grade inflammation, and endothelial dysfunction, all of which are known contributors to microvascular damage in patients with diabetes [36]. Adiposity is a key driver of systemic insulin resistance and is characterized by chronic low-grade inflammation, characterized by elevated circulating levels of pro-inflammatory mediators such as interleukin-6, tumor necrosis factor-α, and C-reactive protein [37]. These factors can disrupt the integrity of the blood–retinal barrier (BRB) by altering tight junction proteins and promoting leukostasis, ultimately increasing vascular permeability [38]. In parallel, obesity-associated dysregulation of adipokines—such as decreased adiponectin and increased leptin levels—further exacerbates endothelial dysfunction and oxidative stress, creating a permissive environment for the accumulation of retinal fluid [39,40]. VEGF, a potent mediator of angiogenesis and vascular permeability, serves as a central effector linking these processes to diabetic maculopathy [41]. In the diabetic retina, hyperglycemia, hypoxia, and inflammation converge to upregulate VEGF expression in retinal cells, including Müller glia and the retinal pigment epithelium [42]. Importantly, both obesity and insulin resistance have been shown to potentiate VEGF signaling, either directly or through intermediary pathways such as those modulated by HIF-1α activation [43]. This amplification of VEGF-driven permeability likely represents a key mechanistic axis through which elevated BMI contributes to the development or progression of macular edema. From a clinical standpoint, these findings suggest that BMI reduction may attenuate VEGF-mediated retinal pathology, positioning weight management as a modifiable upstream target in diabetic maculopathy. Patients with diabetes and elevated BMI should be considered for closer ophthalmologic surveillance and more aggressive metabolic intervention.

Although previous observational studies have reported inconsistent associations between BMI and DME [11,44,45,46], the present MR findings provide evidence that is less susceptible to confounding factors and reverse causation, strengthening the argument for a causal relationship. These findings are supported by a previous MR study that demonstrated that having a higher BMI was a causal risk factor for diabetic complications [for diabetic retinopathy, OR = 1.33 (95% CI: 1.22–1.45); for diabetic nephropathy, OR: 1.74 (95% CI: 1.47–2.07), with significant effect sizes that remained robust even after adjustment for glycated hemoglobin [45]. Consistent with the present findings, prior MR studies have also reported a significant positive causal association between genetically predicted BMI and proliferative diabetic retinopathy, with robust effect estimates (OR = 1.120 (95% CI: 1.076–1.167), *p* < 0.001) [47].

Unlike the well-established impact of BMI, relatively little is understood about the more complex role of IGF-1 in diabetic maculopathy development. As a growth factor that is structurally homologous to insulin, IGF-1 exerts pleiotropic effects on endothelial cells, including promoting cell survival and proliferation while also increasing vascular permeability [48,49]. Experimental studies have suggested that IGF-1 can upregulate VEGF expression and enhance VEGF receptor signaling, thereby potentiating angiogenic responses [50,51]. Moreover, IGF-1 signaling intersects with the PI3K/Akt pathway, which plays a critical role in regulating nitric oxide production in endothelial cells and maintaining vascular homeostasis [51,52]. Thus, in the diabetic retina, dysregulated IGF-1 activity may contribute to the breakdown of the BRB, both directly and indirectly via VEGF-mediated mechanisms [13]. Endothelial cells are ensheathed by pericytes and the foot processes of Müller cells in the BRB, and their breakdown results in enhanced vascular permeability, which is a hallmark of the progression of diabetic maculopathy [53]. IGF-1 has been shown to contribute to BRB dysfunction, the development of retinal microvascular abnormalities, and neovascularization via multiple pathways, including VEGF, dopamine, mitogen-activated protein kinase/extracellular signal-regulated kinase, and c-Jun N-terminal kinase signaling pathways [13]. Furthermore, IGF-1 has been shown to exert mitogenic effects on microglia, suggesting its involvement in their activation and proliferation. Collectively, these findings indicate that IGF-1 may modulate retinal inflammatory processes in diabetic maculopathy [54].

In this study, the differential statistical significance observed for IGF-1 across the MR analysis methods may reflect underlying biological heterogeneity or pleiotropic effects of the selected genetic instruments. Given the evidence of heterogeneity and potential horizontal pleiotropy, MR-PRESSO was selected as the primary analytical approach in this study. The consistency of the BMI results across both the MR-PRESSO and IVW methods enhanced the robustness of the findings. In contrast, the discrepancy observed between the analytical methods for IGF-1 highlights the need for cautious interpretation and suggests that further investigation using larger datasets or more refined genetic instruments may be warranted.

The present findings have important clinical and translational implications. While anti-VEGF therapies remain the cornerstone of treatment for DME, a substantial proportion of patients exhibit suboptimal or incomplete responses, highlighting the need to better understand the upstream drivers of the disease. The results suggest that systemic metabolic factors—particularly obesity and insulin resistance—may modulate retinal VEGF activity and inflammatory signaling, thereby influencing both the onset of the disease and the therapeutic response. In terms of patient management, these findings support the incorporation of BMI assessment into routine diabetic eye care, as elevated BMI may identify patients at heightened risk for diabetic maculopathy who would benefit from closer ophthalmologic surveillance and earlier intervention. For risk stratification, patients with diabetes presenting with concurrent obesity or metabolic syndrome should be considered a high-priority subgroup warranting intensified monitoring and proactive metabolic optimization. Furthermore, integrated management strategies targeting upstream metabolic dysfunction—including lifestyle modification and pharmacological agents such as GLP-1 receptor agonists or SGLT-2 inhibitors—may enhance treatment efficacy, reduce disease incidence, and potentially improve responsiveness to anti-VEGF therapy, representing a promising avenue for future prospective investigation.

This study has several notable strengths, including its use of a two-sample MR design to mitigate the effects of confounding factors and reverse causation, the integration of large-scale GWAS datasets, and the application of multiple sensitivity analyses to assess the robustness of the findings. However, this study has several notable limitations that must be considered when interpreting the findings. Most critically, the two-sample MR design relies on GWAS summary statistics derived from two separate cohorts, both of European ancestry. While the use of ancestry-matched populations mitigates some confounding, European populations are not genetically homogeneous, and meaningful genetic heterogeneity exists across European subgroups. Drawing causal inferences across two datasets that may differ in population structure, linkage disequilibrium patterns, and environmental exposures introduces assumptions that cannot be fully verified in the present analysis. In the most conservative interpretation, this cross-cohort design represents a potential source of systematic bias that could affect the validity of the causal estimates reported here. Second, the analysis was restricted to individuals of European ancestry. Although this restriction may reduce bias due to population stratification, it limits the generalizability of our findings to non-European populations, in whom genetic architecture, allele frequencies, LD patterns, environmental exposures, and disease risks may differ. Therefore, further studies using large-scale GWAS data from diverse ancestral populations are warranted. Although both the exposure and outcome GWAS datasets were restricted to individuals of European ancestry, the UKB and FinnGen populations may not be fully comparable. The Finnish population may be more genetically homogeneous and may differ from the UK population in terms of founder effects, allele frequencies, LD patterns, demographic history, and immigrant population composition. These differences may affect the transportability of genetic associations across datasets and may introduce uncertainty into the MR estimates. Therefore, our findings should be interpreted with caution and require replication in additional European and non-European populations. Third, although heterogeneity and horizontal pleiotropy were assessed using Cochran’s Q test, Rücker’s Q statistic, MR-PRESSO, and MR-Egger regression, significant heterogeneity was observed. This suggests that SNP-specific causal estimates were not fully consistent and that some instruments may have influenced diabetic maculopathy through pathways other than the exposure of interest. Although the MR-Egger intercept did not provide clear evidence of directional pleiotropy, residual pleiotropy cannot be completely excluded. Such heterogeneity or residual pleiotropy may have biased the MR estimates either away from or toward the null. Therefore, the observed associations, particularly those showing heterogeneity or method-specific inconsistency, should be interpreted with caution. Furthermore, the use of summary-level data precludes the ability to conduct stratified analyses by disease severity or specific phenotypic subtypes, such as center-involving DME. Finally, the effect estimates reflect lifelong genetic exposure and may not directly correspond to the impact of short-term clinical interventions. Fourth, BMI is a composite exposure reflecting multiple metabolic perturbations, and lipid-related mechanisms—including dyslipidemia-driven endothelial dysfunction and retinal lipid deposition—may independently contribute to diabetic maculopathy beyond insulin resistance alone. Future MR studies employing lipid-specific genetic instruments are needed to disentangle these parallel pathways. Fifth, although our findings provide genetic evidence for a causal role of elevated BMI, they should be regarded as hypothesis-generating. The MR design precludes direct inference regarding the effect of intentional weight loss on disease outcomes, and prospective randomized trials evaluating weight reduction interventions in diabetic maculopathy are warranted before formal preventive recommendations can be made. Lastly, the present study does not address the mechanistic duality of IGF-1 in the retinal microenvironment. While circulating IGF-1 has been implicated in both neuroprotective and pro-angiogenic processes, the relative balance between these opposing roles—and how this balance is perturbed in the context of diabetic maculopathy—cannot be determined from the current MR framework. Furthermore, as this study assessed systemic blood IGF-1 levels rather than intraocular IGF-1 concentrations, the findings may not fully capture the local retinal dynamics of IGF-1 signaling. Future studies specifically designed to investigate the compartment-specific and context-dependent effects of IGF-1 in the diabetic retina are warranted.

## 5. Conclusions

Ultimately, this study provides compelling genetic evidence that elevated BMI plays a causal role in the development of diabetic maculopathy, and the effects are likely mediated through intertwined inflammatory and VEGF-dependent pathways. These findings highlight the retina as a metabolically sensitive tissue and support a model in which systemic metabolic dysregulation drives local microvascular pathology. Targeting upstream metabolic and inflammatory pathways may represent a promising avenue for the prevention and treatment of DME. Regarding IGF-1, the association with diabetic maculopathy was not consistent across MR methods, reaching nominal significance only in MR-PRESSO. This finding should therefore be considered exploratory and hypothesis-generating rather than confirmatory evidence of a causal relationship. Whether IGF-1 modulates angiogenic signaling in this context remains uncertain, and further validation in independent prospective studies is warranted before any clinical implications can be drawn.

## Figures and Tables

**Table 1:** Summary statistics of data sources.

Traits	Data Source	No. of Participants	Population	No. of Variants	Reference
BMI	UKB	413,186	European	23,079,730	Pan-UK Biobank (https://pan.ukbb.broadinstitute.org, accessed on 20 February 2026)
IGF-1	UKB	398,797	European	23,000,871	
Diabetic maculopathy	FinnGen	86,890 (4603 cases + 82,287 controls)	European	21,253,122	https://finngen.gitbook.io/documentation/data-download, accessed on 23 February 2026

BMI, Body mass index; IGF-1, Insulin-like Growth Factor 1; UKB, UK Biobank.

**Table 2:** Heterogeneity and horizontal pleiotropy of instrumental variables.

Exposure				Heterogeneity		Horizontal Pleiotropy				
							MR-Egger		MR-Egger (SIMEX)	
	N	F	*I*^2^ (%)	P *	P ^#^	P ^†^	Intercept, β (SE)	P	Intercept, β (SE)	P
BMI	467	61.31	86.39	<0.001	<0.001	<0.001	−0.0036 (0.0037)	0.330	−0.0044 (0.0041)	0.288
IGF-1	443	107.01	96.20	0.005	0.006	0.006	0.0027 (0.0026)	0.299	0.0027 (0.0027)	0.309

*: Cochran’s Q test from IVW, ^#^: Rücker’s Q’ test from MR-Egger, ^†^: MR-PRESSO global test, β, beta coefficient; BMI, Body mass index; F, mean F statistic; IGF-1, Insulin-like Growth Factor 1; IVW, inverse-variance weighted; MR, Mendelian randomization; N, number of instruments; PRESSO, Pleiotropy RESidual Sum and Outlier; SE, standard error; SIMEX, simulation extrapolation.

## Data Availability

The datasets used and/or analyzed in the current study are available from the Pan-UK Biobank (https://pan.ukbb.broadinstitute.org/downloads/index.html, accessed on 20 February 2026) and FinnGen (https://finngen.gitbook.io/documentation/data-download, accessed on 23 February 2026).

## Supplementary Materials

The following supporting information can be downloaded at: https://www.mdpi.com/article/10.3390/biomedicines14061178/s1, Table S1: List of single-nucleotide polymorphisms used as instrumental variables in MR analysis.

## Abbreviations

The following abbreviations are used in this manuscript:

Akt	Protein kinase B
AP-1	Activating protein 1
BMI	Body mass index
BRB	Blood–retinal barrier
CI	Confidence interval
DM	Diabetes mellitus
DME	Diabetic macular edema
GWAS	Genome-wide association study
HIF-1α	Hypoxia-inducible factor 1 alpha
IGF-1	Insulin-like growth factor 1
IVs	Instrumental variables
IVW	Inverse-variance-weighted
LD	Linkage disequilibrium
MR	Mendelian randomization
NF-κB	Nuclear factor kappa B
OR	Odds ratio
PI3K	Phosphoinositide 3-kinase
PRESSO	Pleiotropy RESidual Sum and Outlier
SE	Standard error
SIMEX	Simulation Extrapolation
SNPs	Single-nucleotide polymorphisms
UKB	UK Biobank
VEGF	Vascular endothelial growth factor
